# Text-Book of Nervous Diseases

**Published:** 1902-04

**Authors:** 


Text-Book of Nervous Diseases. Being a Compendium for the use of Students and Practitioners of Medicine. By Charles L. Dana, A. M, M. D., Professor of Nervous Diseases in Cornell University Medical College, etc. Fifth edition, with 244 illustrations. Pp. xiii.—633. New York: William Wood & Co. 1901.

A new edition of this work is always welcomed. In this instance we find the book is practically like the fourth edition, with the exception that there is an excellent chapter upon general paresis. The illustrations are numerous and extremely helpful.

There is a clearness in Dana's descriptions which makes the treatise a great favorite with students and teachers. We believe that one of the most practical chapters is the one on neurological therapeutics. The author considers in detail special applications of hydrotherapy, electro-therapeutics, vibratory therapeutics, suspension, organic extracts, the rest cure, the Fränkel movements, lumbar puncture, and the use of the newer drugs. The text-book is in every way satisfactory. J.W.P.

